# Inferring species interactions from ecological survey data: A mechanistic approach to predict quantitative food webs of seed feeding by carabid beetles

**DOI:** 10.1002/ece3.8032

**Published:** 2021-08-25

**Authors:** Michael J. O. Pocock, Reto Schmucki, David A. Bohan

**Affiliations:** ^1^ UK Centre for Ecology & Hydrology Wallingford, Oxfordshire UK; ^2^ Agroécologie, AgroSup Dijon INRAE, Université de Bourgogne Franche‐Comté Dijon France

**Keywords:** biocontrol, Carabidae, ecological network, predation, seed rain, weighted interaction

## Abstract

1. Ecological networks are valuable for ecosystem analysis but their use is often limited by a lack of data because many types of ecological interaction, for example, predation, are short‐lived and difficult to observe or detect. While there are different methods for inferring the presence of interactions, they have rarely been used to predict the interaction strengths that are required to construct weighted, or quantitative, ecological networks.
2. Here, we develop a trait‐based approach suitable for inferring weighted networks, that is, with varying interaction strengths. We developed the method for seed‐feeding carabid ground beetles (Coleoptera: Carabidae) although the principles can be applied to other species and types of interaction.
3. Using existing literature data from experimental seed‐feeding trials, we predicted a per‐individual interaction cost index based on carabid and seed size. This was scaled up to the population level to create inferred weighted networks using the abundance of carabids and seeds from empirical samples and energetic intake rates of carabids from the literature. From these weighted networks, we also derived a novel measure of expected predation pressure per seed type per network.
4. This method was applied to existing ecological survey data from 255 arable fields with carabid data from pitfall traps and plant seeds from seed rain traps. Analysis of these inferred networks led to testable hypotheses about how network structure and predation pressure varied among fields.
5. Inferred networks are valuable because (a) they provide null models for the structuring of food webs to test against empirical species interaction data, for example, DNA analysis of carabid gut regurgitates and (b) they allow weighted networks to be constructed whenever we can estimate interactions between species and have ecological census data available. This permits ecological network analysis even at times and in places when interactions were not directly assessed.

## Graphical abstract

Ecological networks are valuable for ecosystem analysis, but often limited by lack of data on interactions between species. Here, we develop a trait‐based approach to infer interaction strengths with mechanistic models and take existing ecological census data (samples of species: of carabid ground beetles and weed seeds) to construct inferred quantitative food webs.

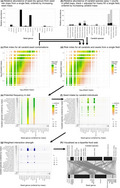

## 1 INTRODUCTION

Networks are a valuable tool for understanding the structure and dynamics of ecosystems (Kaiser‐Bunbury & Blüthgen, 2015; Ma et al., 2019; Pocock et al., 2012; Tylianakis et al., 2010). Ecological networks are constructed from information on biotic interactions, for example, species–species interactions, and provide a whole system approach to understanding changes in biodiversity, the resilience of biodiversity to environmental change, and the provision of ecosystem function (Heleno et al., 2014). Weighted networks are especially valuable in ecological network analysis because many ecosystem functions are influenced by interaction frequency or importance (Kaiser‐Bunbury & Blüthgen, 2015). Despite the value of a network approach to studying ecological systems, we are often severely limited by the lack of empirical data on biotic interactions: This has been termed the “Eltonian shortfall” (Hortal et al., 2015). In the past, researchers have sought to address this shortfall by using different methods to infer the presence of interactions from data on species presence, but it would be valuable to have methods to infer quantitative interactions and so gain the benefits from using weighted network analysis.

The lack of data on species interactions is because many interactions are relatively brief (e.g., an animal consuming an arthropod) or cryptic (e.g., predation occurring at night or underground). This means that interactions are more difficult to sample than species. As a consequence, many ecological networks studies are based on (a) interactions that are comparatively easy to record, for example, direct observations of pollinating insects visiting flowers (Memmott, 1999) or analysis of gut contents of “gulp predators” such as fish (Gray et al., 2014); or (b) interactions that are long‐lasting, for example, parasitoids and their hosts (Müller et al., 1999) or phytophagous insects and their food plants, both of which can be identified using rearing or DNA‐based methods (Evans et al., 2016). Even in these examples, adequate sampling of the network of interactions is far more costly (in time and/or resources) than sampling the organisms alone (Banašek‐Richtera et al., 2004). This means that many types of biotic interaction are under‐represented in ecological research, despite being of interest and high functional importance.

There are many datasets where assemblages of potentially interacting species (e.g., predators and prey) have been sampled using standard ecological census techniques, but since the interactions themselves have not been sampled, the data cannot be used for network analyses. If it was possible to infer these interactions, these datasets would provide a vast resource about changes in (inferred) networks over time and space (Delmas et al., 2019) and hence on ecosystems and their dynamics. The inference of biotic interactions is an active area of research, and broadly four approaches have been used in the past.

1. *Using databases of previously recorded interactions* and applying these to datasets of co‐occurring species. This relatively simple approach has been used for a range of taxa (Gray et al., 2014; Muñoz et al., 2019; Redhead et al., 2018), and it makes the assumption that if two species were reported as interacting at one time and place, then they will always interact when they co‐occur. This also depends on the availability and comprehensiveness of the information on recorded interactions (Poelen et al., 2014). Databases could be biased toward common interactions, because they are most frequently observed, or toward uncommon interactions, because they are most frequently reported (because the observer deems them to be noteworthy).
2. *Co‐occurrence analysis* is used to statistically define associations from samples obtained at the same time and place. This is particularly useful for datasets of many discrete samples, for example, DNA meta‐barcoding of species from environmental samples (Lima‐Mendez et al., 2015; Vacher et al., 2016) or gene regulatory networks from gene‐expression microarray data (Marbach et al., 2012). It has been applied to biodiversity data, where citizen science provides a large number of co‐occurrence records (Milns et al., 2010). One challenge with this approach is that when an association does occur, further information is required to determine the type of interaction (Faust & Raes, 2012; Freilich et al., 2018); for example, an association between two species could be due to predation, mutualism, or shared resource use.
3. *Inductive machine learning approaches* can be computationally‐ and data‐intensive but only require simple rules to be provided (e.g., small species are preyed upon by larger species) and the networks are learned from data. For example, inductive machine learning has been used with time series of species abundance data from agroecosystems to successfully identify previously under‐recorded predatory interactions, namely: spiders predating small carabid ground beetles (Bohan et al., 2011).
4. *Trait‐based approaches* have been used when there is an allometric relationship between size of predator and prey (Beckerman et al., 2006; Gravel et al., 2013; Petchey et al., 2008; Sebastián‐González et al., 2017; Williams et al., 2010), for example, when gape size limits prey selection. They can be used for a range of other interaction proxies, such as phylogenetic or niche distance (Morales‐Castilla et al., 2015).

These inference approaches have different benefits, depending on the datasets available and the ambitions of the researchers (Faisal et al., 2010). However, the inferred networks are typically presented as presence (or probability) of interactions, rather than weighted interactions. There are many different ways of defining interaction strength that are relevant for different purposes; here, we focus on frequency of consumption (Berlow et al., 2004). In general, quantifying the weights, or strengths, of interactions is valuable. This is because (a) knowledge about the distribution of strong and weak links helps us understand network stability (Bascompte et al., 2006; Jordano, 1987); (b) measures of interaction strength provide information on the functional importance of interactions (Vazquez et al., 2007); and (c) there is a practical benefit that weighted network analysis is more robust to sampling biases than unweighted network analysis because the analysis is more influenced by interactions with higher weights (e.g., frequent interactions) than by rare, stochastic interactions (Bersier et al., 2002).

Here, we considered weed seeds in arable fields and their predation by carabids (ground beetles, Coleoptera: Carabidae). Carabids are important multifunctional predators in agroecosystems (Honek et al., 2003; Ma et al., 2019). Many are important seed feeders with seeds either as part or all of their diet, depending on the species (Honek et al., 2003; Kulkarni et al., 2015). Practically, seed‐feeding carabids provide a valuable ecosystem service in helping to regulate weed seeds in arable fields (Bohan et al., 2011; Petit et al., 2018), so justifying frequency of consumption as a meaningful measure of interaction strength. While carabids and seeds can be sampled relatively straightforwardly with standard ecological census techniques (Brooks et al., 2003; Heard et al., 2003), it is much harder to detect their seed‐feeding interactions. Here, we develop a mechanistic model of prey preference (frequency‐dependent foraging) to infer weighted interactions of carabid ground beetles preying upon weed seeds at the soil surface of arable fields and apply it to a large dataset of weed seed and carabid abundances.

## 2 METHODS

### 2.1 Developing a mechanistic trait‐based model to infer weighted food webs

**FIGURE 1:**
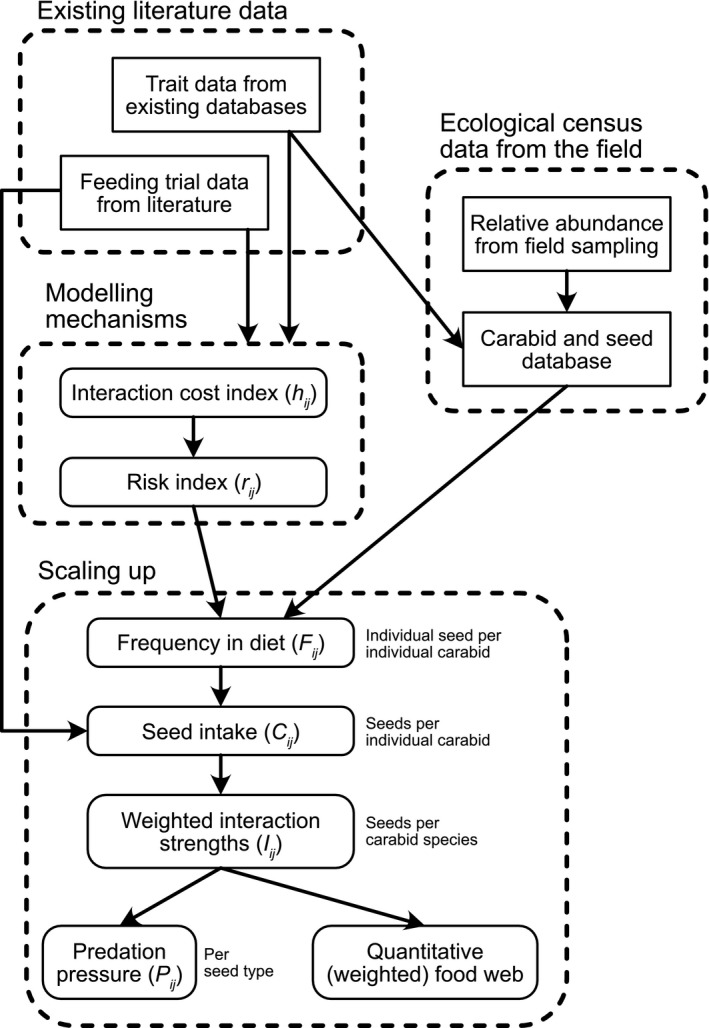
Summary of the process to use data from ecological sampling and from the literature to scale up to inferred food webs

The fundamental variable needed to construct weighted ecological interaction networks is the frequency of each prey in the diet of each predator present in the field (*F_ij_*). This relies on predicting the foraging behavior and prey selection of the predators. There is a rich literature about foraging theory and predator–prey dynamics, which has also been used to predict food web complexity (Beckerman et al., 2006; Petchey et al., 2008). In the case here, we needed a model of prey selection based on *relative* search and handling time so that we could use data from empirical feeding trials in the literature. (In contrast, optimal foraging models typically require absolute values for these parameters (Pyke et al., 1977).) The model for frequency‐dependent prey selection, as described by Gendron (1987), fitted this requirement so we used that to estimate the frequency of each seed in the diet of each carabid (*F_ij_*), and scaled this up to estimate the interactions for a whole inferred network based on empirical data on seed and carabid density in arable fields. This process is described in detail below (Figure 1; Table 1).

**TABLE 1:** Parameters in the conversion of risk indices to interaction strengths for construction of weighted food webs of seed‐feeding carabids

Parameters	Description	Seed	Carabid	Directly comparable across carabid species?	Calculation
Interaction cost index (*h_ij_*)	An estimate of the total costs, including inherent preference, handling time, and other costs, for carabid *j* on seed *i*	Per individual	Per individual	No	Derived from analysis of data in the literature (see text)
Risk index (*r_ij_*)	Measure of the risk index, based on species traits (see text, where *E_i_* is the energy content of a single seed). Called profitability by other authors when *h* refers strictly to handling time.	Per individual	Per individual	No	$r_{\mathrm{ij}}=\frac{E_{i}}{h_{\mathrm{ij}}}$
Frequency in diet (*F_ij_*)	Risk index multiplied by seed abundance (*D_i_*), normalized as the proportion in the diet per carabid species	Per site	Per individual	No	$F_{\mathrm{ij}}=\frac{r_{\mathrm{ij}}D_{i}}{\sum_{j}r_{\mathrm{ij}}D_{i}}$
Seed intake per carabid (*C_ij_*)	Expected frequency in diet (*F_ij_*) scaled by energetic intake per carabid individual (where *E_j_* is the energetic requirement of an individual carabid)	Per site	Per individual	Yes	$C_{\mathrm{ij}}=F_{\mathrm{ij}}\times \frac{E_{j}}{\sum_{i}\left(F_{\mathrm{ij}}E_{i}\right)}$
Weighted interaction strength (*I_ij_*)	Seed intake multiplied by carabid relative abundance (*D_j_*)	Per site	Per site	Yes	$I_{\mathrm{ij}}=C_{\mathrm{ij}}D_{j}$
Predation pressure (*P_i_*)	Sum of weighted interaction strengths for a seed relative to the seed abundance	Per site	n/a	n/a	$P_{i}=\frac{\sum_{j}I_{\mathrm{ij}}}{D_{i}}$

Note

In our example, we consider *i* = seed genus and *j* = carabid species.

In the model of frequency‐dependent prey selection, the frequency (*F_i_*) of a prey item *i* in the diet of a predator is the normalized function of its “risk index” (*r_i_*) and its density (*D_i_*) (Table 1). The calculation of *F_ij_* therefore requires calculation of the risk index (*r_ij_*), which itself requires estimation of an interaction cost index (*h_ij_*). The definition of the risk index as *E*/*h* is the same as “profitability” in the optimal foraging model (Tinbergen, 1960). However, here we use the parameter *h* to define the totality of the “costs” (handling time, innate preference of the predator for the prey, and so on) and so, hereafter, we refer to *h* as the “interaction cost index” (rather than the “handling time” sensu stricto) and *r* as the “risk index.” If the risk index is density‐dependent, then changes in prey density will lead to prey switching (Gendron, 1987; Kondoh, 2003; Manly et al., 1972), but here we consider the simple case of no‐switching, where *r_i_* is density‐independent.

The data we used to calculate the interaction cost index (*h*) were obtained from published “cafeteria experiments,” which are choice experiments in which the consumption of prey by predators is tested in experimental arenas. These have been used to assess carabid prey preferences for seeds (Honek et al., 2007; Petit et al., 2014) and invertebrates (Lang & Gsödl, 2008). We obtained data from cafeteria experiments for two species of carabid feeding on 64 species of seed (Honek et al., 2003) and for five species of carabid feeding on ten species of seed (Petit et al., 2014). In these studies, individual beetles were provided with seeds of seven (Honek et al., 2003) or ten (Petit et al., 2014) different species of seed in experimental arenas. Researchers daily recorded the number of seeds that had been consumed on a daily time step. For Honek et al. (2003), the data on time to remove 50% of seeds (CT_50_) were digitized from Figure 4 in and converted to a daily consumption rate (*x_i_*): *x_i_* = (0.5 × number of seeds used in the experiment)/CT_50_.

We assumed that the number of seeds consumed was proportional to the “risk index” of that seed species for that carabid species (*x_i_* ∝ *r_i_*). We combined this with the definition of the risk index: *r_i_* = *E_i_*/*h_i_* (Tinbergen, 1960). By re‐arrangement, we can define the following: *h_i_* = *E_i_*/*x_i_*, which means that *h_i_* was comparable within, but not between, different experiments. To model *h*, we then used predator and prey size because these traits are major determinants of weed seed preferences (Kulkarni et al., 2015). There are many possible alternative models for the allometric handling time relationship between predators and prey, which variously include predator and/or prey size (Petchey et al., 2008). Here, we chose to include predator size, prey size, and their interaction. Initial investigation indicated it was appropriate to model the interaction cost index (*h_ij_*) as a function of the interaction of seed size (cube root of seed mass) and carabid size (log‐transformed body mass), and we included the study as a random effect:

$$\log_{e}\left(h_{\mathrm{ij}}\right)=\beta_{0}+\beta_{1}\sqrt[{3}]{M_{i}}+\beta_{2}\log_{e}\left(M_{j}\right)+\beta_{3}\left(\sqrt[{3}]{M_{i}}\times \log_{e}\left(M_{j}\right)\right)+{\text{Study}}_{k}$$

There were few very large seeds (>5 mg), but their predicted interaction costs were high so we weighted the data points by the inverse of the cube root of seed mass (=1/*M_i_* ^1/3^) to reduce their influence on the results. To calculate the risk index for each carabid and seed combination, we used the interaction cost index and the energetic content of the seed (Table 1). By using a trait‐based approach to estimate *h* from data in the literature, this meant that we could estimate the risk index (*r*) for carabid and seed species (based on their mass) even if they were not included in the published cafeteria experiments.

Having obtained the risk index for each carabid‐seed combination, we scaled this by the density of the seeds (*D_i_*) and normalized it to calculate the predicted frequency (*F_ij_*) of seed *i* in the diet of an individual carabid of species *j*. We then needed information on the energy intake of carabids in different feeding guilds. Since the seed predators in this study were all closely related carabid species, they are likely to have the same energetic intake (per gram of body mass) of seeds, once their feeding guild was taken into account. Several sources were used to categorize British carabid species into three distinct feeding guilds (Brooks et al., 2012; Harvey et al., 2008; Luff, 2007): Species in Tribes Harpalini and Zabrini were classed as granivorous; species in Tribe Sphodrini, Tribe Trechini, and genera *Pterostichus* and *Poecilus* (from Tribe Pterostchini) and *Agonum* (from Tribe Platynini) were classed as omnivorous; and all other species were classed as obligate carnivores. Published data provided information on the daily energy intake of seeds (mg of seeds per mg carabid body mass). (Note that these are different experiments to the choice experiments used to estimate the handling costs, explained above.) In Honek et al. (2003; their Table 2), 23 species of carabid were given *Cirsium arvense* (1.3 mg) and *Capsella bursa‐pastoralis* (0.1 mg) ad lib. in separate experiments; we used data for the seed with the maximum consumption rate. In Petit et al. (2014; their Table 1), five species of carabid were each given ten species of seed (0.1–8.9 mg) together in a cafeteria experiment. We totaled the daily mass of seeds consumed and modeled the average feeding rate (in mg seeds per mg body mass) for species in the different guilds with a mixed‐effects model with feeding guild as a fixed effect, and species and source of data as random effects.

We scaled up the literature‐derived average feeding rate to the per‐individual energetic requirement of carabids (i.e., *E_j_* = feeding rate (mg per mg) × *M_j_*) and then converted this from the *mass* of seeds to the *number* of seeds of type *i* to give the seed intake per carabid (*C_ij_* = number of seeds of species *i* consumed by an individual of carabid *j*). We then multiplied this by the abundance of carabids of species *j* to give the weighted interaction strength (*I_ij_*) and so combined these to construct a weighted, inferred food web. Finally, we calculated a predation pressure ratio (*P_i_*) for each seed type in each field to provide an assessment of the predicted intensity of seed predation based on the inferred food web.

### 2.2 Applying the inference of interactions to ecological census data of weed seeds and carabids in arable fields

We applied this analysis pipeline to construct inferred food webs with data on seed and carabid abundance from 255 arable fields (see http://doi.org/10.5281/zenodo.4252783 for R code and data to replicate this analysis). The data we used were obtained from the Farm Scale Evaluation (FSE) of genetically modified crops. The FSE was a split‐field experiment comparing the impact of four genetically modified crops to the same crops cultivated conventionally (Firbank et al., 2003). The 255 fields (67 fields of spring oilseed rape, 65 of winter oilseed rape, 66 of beet, and 57 of maize) were distributed across the UK according to where the crops are typically grown (Bohan et al., 2005; Champion et al., 2003). Here, we used the data from the conventionally cultivated half of the fields. Biodiversity surveys were undertaken during the FSE during 2000–2003, and they included seed rain traps to estimate the abundance of soil‐surface seeds and pitfall traps to sample soil‐surface active invertebrates including carabid ground beetles (Brooks et al., 2003; Heard et al., 2003). We used data from all the traps away from the field edge, that is, >2 m from the field boundary. Seed rain traps were 10 cm diameter pots placed 32 m into the field in four locations, and contents were collected every 2 weeks from the anthesis of the first weed species until harvest of the crop (Heard et al., 2003). The seed rain was quantified as the sum of viable seeds from all traps over this period to give the density of the total of seeds available at the soil surface during that season (*D_i_*). Not all seeds could be identified to the species level, so we aggregated all seeds at the genus level. The invertebrate pitfall traps were 6‐cm pots placed along four transects at 8 m and 32 m into the field for two‐week periods. For consistency with the data from the seed rain traps, we only considered invertebrate counts obtained during the growing season of the crops (April/May and June/July for winter‐sown crops and July/August for spring‐sown crops). Carabids from the pitfall traps were identified to the species level.

In our pipeline, we needed data on the mass of seeds and carabids and the energy content of seeds to construct inferred food webs. We used an existing dataset to obtain data on seed mass for all species and for seed energy content for most species (Gibbons et al., 2006). Of the 82 genera of seeds in the FSE dataset, we had information on seed energy content (kJ/g) of species in 60 genera and averaged this across species within each genus. For the 22 genera without this information, we used the average energy content across genera (18.77 kJ/g, interquartile range: 16.04–21.03 kJ/g), although initial exploration indicated that using the average did not make substantial differences to the results. Carabid body mass was derived from body length. The body length for each of the 91 carabid species was calculated as the geometric mean of the maximum and minimum length (in mm) reported by Luff (2007) and was converted to body mass (*M_i_*; dry mass in mg): log_e_(*M_j_*) = −3.4 + (2.6 × log_e_(body length) (Jarosik, 1989; Saska et al., 2019).

Carabid pitfall data are a measure of activity‐density, that is, a combination of density and activity (Thomas et al., 2006), rather than true density. Carabid activity is affected by many factors, including temperature, vegetation cover and body size (because large beetles walk further in a set time and so are more likely to encounter the pitfall trap). The relationship with body size is complex and not easy to predict (Halsall & Wratten, 1988), but recently Engel et al. (2017) used allometric scaling to predict true carabid density (*D_j_*) from abundance in pitfall traps (*n_j_*) and body mass (*M_j_*). The mass‐specific correction factor (*β*) was related to temperature and the arrangement of the traps. Here, we used the correction factor for a single pitfall trap at 21℃ = −0.51 (range: −0.55 to −0.45 for 15 to 27℃) to estimate the relative density of carabids from the pitfall trap data, so *D_j_* = *n_j_* × *M_j_^‐0.51^*. Therefore, for equal numbers of small (2 mg) and large (30 mg) carabids in a pitfall trap, the true density of the small carabids is four times greater than that of the large carabids.

Having constructed inferred weighted food webs for the data from 255 fields, we demonstrated the potential of this approach to be used in ecological network analysis. Firstly, we calculated the weighted connectance of the networks and tested for an effect of crop type, and including the effect of species richness and abundance of seeds and carabids as covariates. Secondly, we tested for an effect of crop type, seed size, and abundance on our new network‐derived metric of predation pressure, with the field as a random effect for intercept and slope to take account of variation in the size and direction of the overall effect.

## 3 RESULTS

### 3.1 Calculation of the risk index

There was a predictable relationship of interaction cost index according to seed and carabid size. The specific result, with data points weighted by the inverse of seed size and the source of the data (Honek et al. (2003) or Petit et al. (2014)) as a random effect, was as follows:

$$\log_{e}\left(h_{i}\right)=‐14.2177+\left(8.739\times \sqrt[{3}]{M_{i}}\right)+\left(1.720\times \log_{e}\left(M_{j}\right)\right)+\left(‐1.278\times \sqrt[{3}]{M_{i}}\times {\text{log}}_{e}\left(M_{j}\right)\right).$$

**FIGURE 2:**
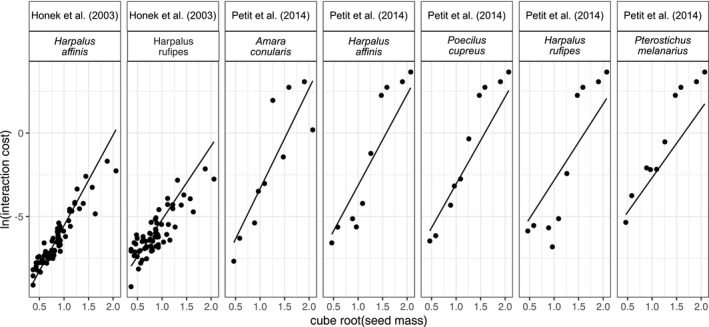
The interaction cost estimated from laboratory studies of seed choice by carabid (ground beetle) species. For each study, carabids are arranged left to right by increasing body size (dry mass for those in Petit et al. (2014) = 8.4, 13.4, 15.6, 29.6, 36.8 mg). Points are the data from published studies (Honek et al., 2003; Petit et al., 2014), and the line shows the fit of the model with seed mass, carabid mass, and their interaction, with data points weighted by the inverse of cube root of seed size

**FIGURE 3:**
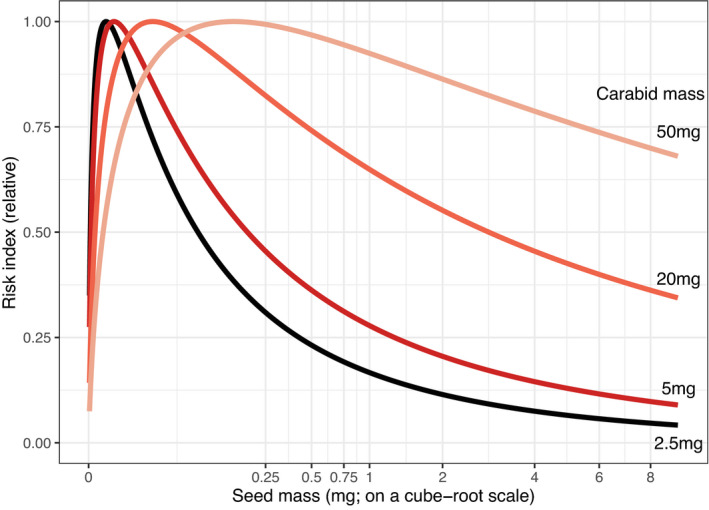
The modeled risk index (*r_ij_*) for seeds and four sizes of carabid (ground beetle). The risk index has been normalized so the maximum for each species is one

This shows that the interaction cost index for large seeds decreased with carabid size, but increased for small seeds (Figure 2). The risk index, calculated by combining the interaction costs with the seed energy content, showed that larger beetles prefer larger seeds and have a wider diet breadth (Figure 3), as previously found in experimental studies (Honek et al., 2003, 2007; Petit et al., 2014).

### 3.2 Estimation of the energetic intake of carabids

**FIGURE 4:**
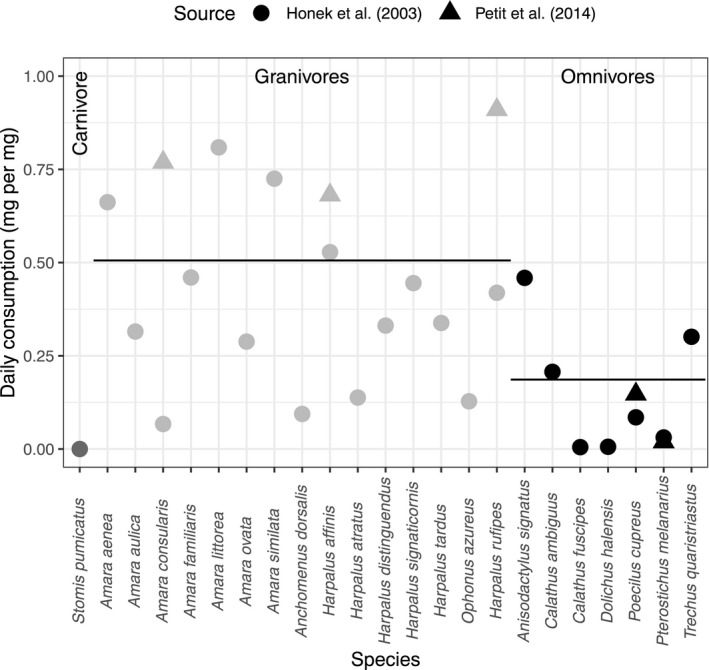
The estimated daily consumption rate of seeds by carabid beetles, from two laboratory studies, varied by their recorded feeding guilds. The horizontal lines show the mean across species in each guild, taking the source of data into account

The daily energy intake reported in experimental studies varied significantly by feeding guild when seeds are provided ad lib. The average daily consumption rates (in mg seed per mg carabid body mass) were 0.51 for granivorous species, 0.18 for omnivorous species, and (as expected) 0.00 for the single carnivorous (Figure 4). The difference between omnivores and granivores was significant: −0.32 ± 0.09 standard error in the mixed‐effects model, showing that omnivorous species ate fewer seeds than similarly sized granivorous species, at least in short‐term experimental studies, even when seeds were the only food available.

### 3.3 Scaling up to a weighted network

**FIGURE 5:**
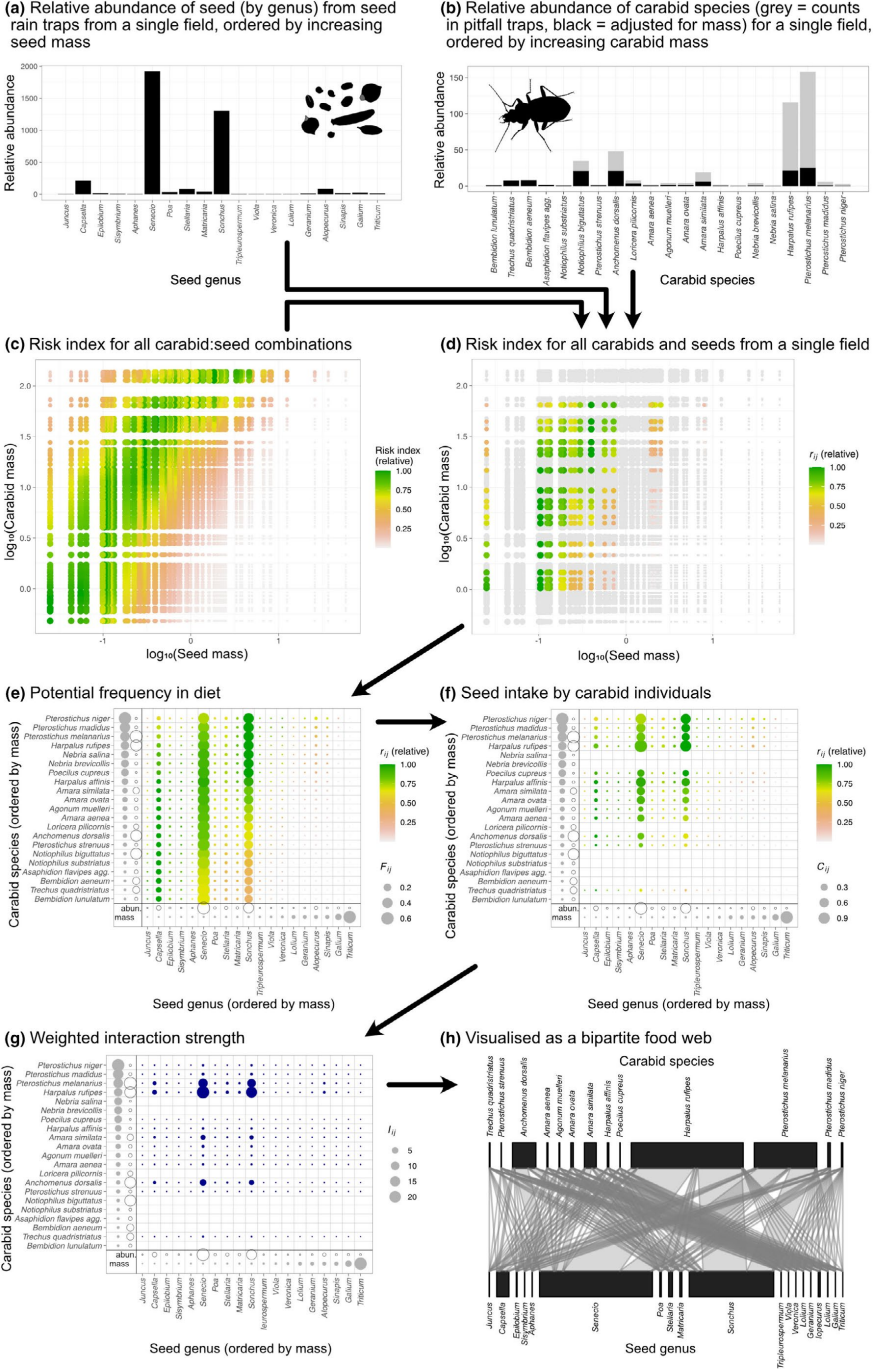
Demonstration of the pipeline for taking the ecological census data for (a) carabids and (b) seeds and, using a predictive model derived from data in the literature to calculate (c) the risk index from every seed:carabid combination, and then taking the risk index values for (d) a single field and scaling them up to (e) potential frequency in diet, (f) seed intake per individual, and (g) weighted species‐species interaction strength, which can be (h) visualized as a bipartite food web. In e, f, and g, the abundance of the carabids and seeds is indicated by open circles and their mass is indicated in the figure margin by gray circles

We scaled up the data to construct the predicted food web for each field in this dataset and exemplified this for a single site (Figure 5; “WR19”: a field that growing winter‐sown oilseed rape, which had the highest number of seed genera + carabid species (19 + 21) out of all sites (median = 17)). The risk index (individual seed‐individual carabid) was calculated for every seed‐carabid combination (Figure 5c). There was slight variation in the risk index between similarly sized seeds because the energy content (kJ/g) varied between seeds. From the subset of seed:carabid combinations of the risk index for this site (Figure 5d), we predicted the frequency of seeds in an individual carabid's diet (Figure 5e), multiplied this by the daily consumption rate (Figure 4) to give the consumption per carabid individual (Figure 5f), and multiplied this by the relative abundance of each carabid species to give the inferred pairwise interaction strength (Figure 5g & h).

**FIGURE 6:**
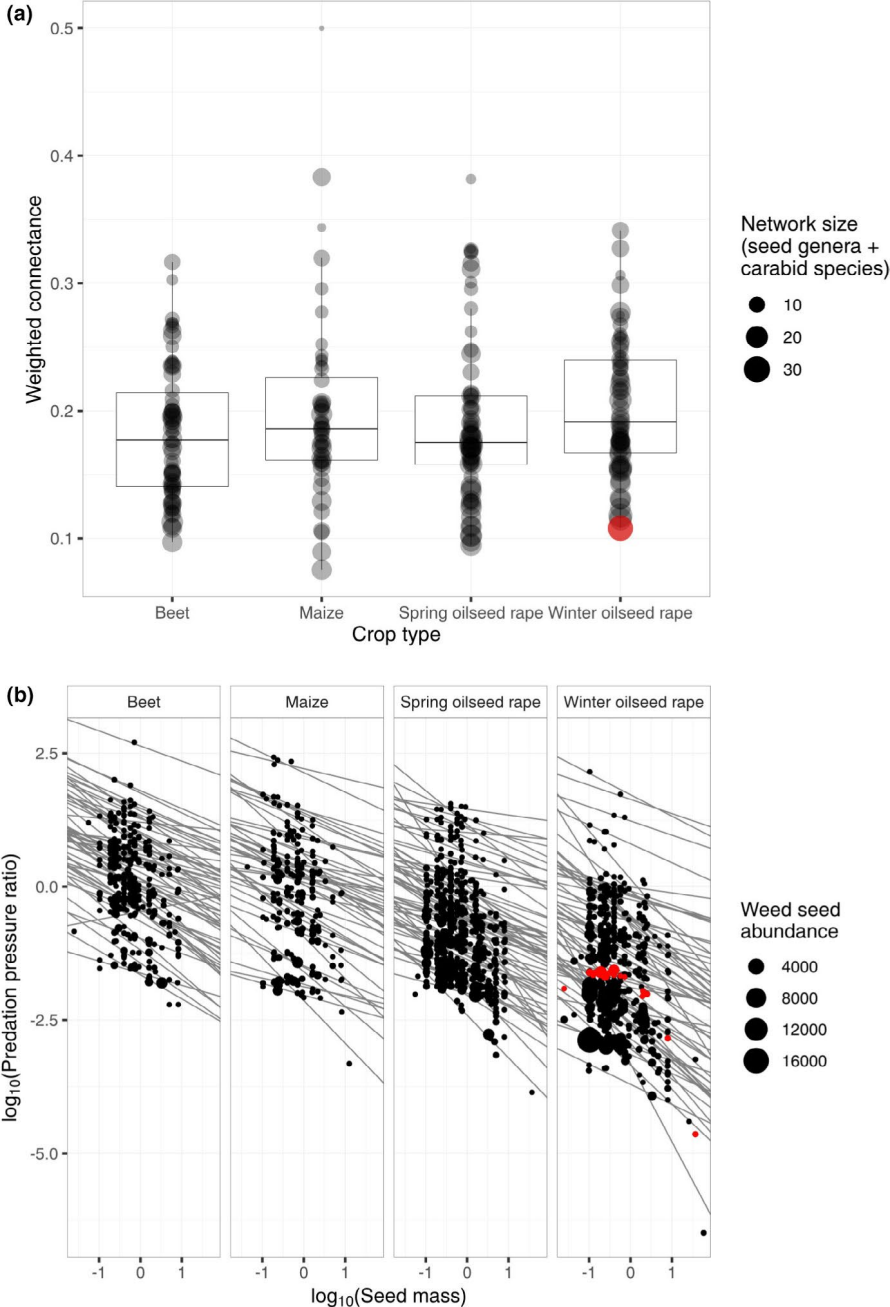
Summary of the inferred carabid‐seed food webs for the 255 fields in the dataset based on (a) weighted connectance of the food web in each field and (b) the predation pressure ratio of each seed type in each field. The red point/s shows the data from field “WR19,” as shown in Figure 5

Inferred networks were constructed for all 255 fields in our dataset in which carabids and seeds were sampled: Ten fields had zero carabids and eight had zero seeds, so were excluded from further analysis. The overall structure of the network, assessed with weighted connectance, was strongly affected by the network size (number of seed genera + number of carabid species; *p* < 0.001) and abundance of seed‐feeding carabids (*p* < 0.001), but not by the field type (*p* = 0.0.95) or the total abundance of weed seeds (*p* = 0.308; Figure 6a). The predation pressure for each seed genus in each field varied substantially: Smaller seeds tended to have higher predation pressure than larger ones (effect size ± standard error (SE) = −0.429 (0.020); Figure 6b), and it varied by the crop type: Beet fields had the highest predation pressure ratio; maize was no different to beet (effect size ± SE compared to beet = −0.293 ± 0.196), spring‐sown oilseed rape was lower (−0.840 ± 0.180 compared to beet), and winter‐sown oilseed rape was lowest (−1.639 ± 0.182 compared to beet). Suitable empirical data are not yet available to validate these models, but these summary trends provide testable hypotheses about the network‐derived ecosystem function of carabids in these fields (Bohan et al., 2021).

## 4 DISCUSSION

One challenge for using ecological network analysis in applied research, such as agroecology or conservation ecology, is the lack of availability of information on interactions. Here, we used a mechanistic approach to infer weighted predator–prey food webs from ecological survey data and species' traits. This mechanistic approach to inferring species interactions has not often been done in the past, although notably Beckerman et al. (2006) did this with allometric functional responses, rather than using foraging data from feeding trials as we did. It was valuable to develop this method for carabid beetles because they are mobile predators of invertebrate pests and weed seeds in arable crops. In particular, they are known to contribute to weed seed regulation in agroecosystems (Bohan, Boursault, et al., 2011; Honek et al., 2003; Kulkarni et al., 2015), and network approaches have been recommended to study the direct and indirect effects of carabid biocontrol (De Heij & Willenborg, 2020). A novelty of our approach, compared to others (Morales‐Castilla et al., 2015), is that we took the further step to predict the interaction strength (here assessed as the frequency of interactions), scaled up from per capita estimates of interactions, rather than simply assessing the presence or probability of interactions. Our approach can be used whenever the risk index for predator–prey interactions can be predicted (e.g., based on trait‐matching) and where ecological census data exist, thus allowing us to create predicted food webs in places and at times when the interactions were not studied.

One key question from this research remains: Are these inferred networks true? It is challenging to answer this question because information on carabid‐seed feeding is often lacking and difficult to obtain from the field. One way of testing this would be to consider the relationship between network structure and ecosystem properties (Ma et al., 2019). Here, we developed a new metric derived from the quantitative network structure: the predation pressure ratio, to explain the predicted predation rate relative to the seed abundance. Recently, we found that the predation pressure ratio was valuable in explaining variation in weed seed dynamics, and more valuable than carabid abundance alone (Bohan et al., 2021). This supports our approach being ecologically meaningful. The importance of this network‐derived metric could be tested in the field, for example, with bioassays of seed consumption via seed cards (Menalled et al., 2000).

An alternative approach is to view our results as a null model that can be tested against species interaction data, when it becomes available. Our models were simple in assuming that there was bottom‐up control of the carabids and assuming no interference in the field due to intraguild competition between carabids for seed resources (Carbonne et al., 2019). Using inferred networks as a null model may show how the mechanistic models need to be refined, for example, including the effect of traits such as seed shape, integument thickness or lipid content (Gaba et al., 2019; Honek et al., 2007; Sebastián‐González et al., 2017), or other local drivers, for example, total seed abundance, vegetative cover, or the presence of alternate invertebrate prey. Competition for alternate prey or density dependence in the risk index would lead to prey switching (Gendron, 1987; Manly et al., 1972), which has recently been inferred for carabids from empirical data (Carbonne et al., 2020; Gray et al., 2021). Therefore, when suitable data are available, each of these assumptions could be tested in future models.

Data that are suitable for directly testing our models on carabid seed‐feeding are likely to become widely available soon via DNA‐based analysis of gut regurgitates of carabids (Sint et al., 2018; Wallinger et al., 2015), and already simple food webs have been constructed from DNA analysis of carabid gut contents (Frei et al., 2019). The integration of such data together with ecological sampling of the abundance of carabids and soil‐surface weed seeds will enable models to be validated and refined, thus providing a test of the underlying mechanisms leading to the assembly of food webs.

The impact of alternate prey would be especially important for the omnivorous carabid species that can switch to prey upon invertebrates such as slugs or aphids (Bohan et al., 2000; Gray et al., 2021), but even for these species, seeds are likely to be a major source of food (Frei et al., 2019). We suggest that the network model we have developed could, with care, be extended to include invertebrates as an alternate prey if the risk index could be estimated for invertebrates as well as seed prey (Roubinet et al., 2018). This would provide a powerful framework for predicting biocontrol across the community of granivorous and omnivorous carabids in agroecosystems (De Heij & Willenborg, 2020; Ma et al., 2019).

In our study, we were able to reuse available data to model the interaction cost index (*h*, and hence risk index *r*; Figure 3) and energetic intake per individual (*E*; Figure 4), and so did not need to undertake new experiments to inform these parameters. Our reanalysis of data from the literature indicated that carabid feeding does broadly adhere to mechanistic expectations (Figures 2 and 3) and our results matched previous qualitative expectations. If these models were extended to other organisms, there may be a lack of data in the literature, although an allometric model of handling time has previously been used to infer bird‐seed feeding interaction strengths (Pocock et al., 2012). If new experiments are required to parameterize the models, it is strongly recommended that standardized experimental approaches should be used (Deroulers et al., 2020), thus enabling results from multiple experiments to be comparable.

We note that optimal foraging theory (Pyke et al., 1977) is an alternative approach to frequency‐dependent prey selection (Gendron, 1987), but optimal foraging models require that its equation denominators are in identical units, for example, handling time and search time in seconds, movement in meters per second, prey density per meters squared. Although it is possible to directly assess the handling time of seeds by carabids (Charalabidis et al., 2017), the data required for optimal foraging theory will be available only in very well‐studied systems or where it can be estimated via allometry (Beckerman et al., 2006; Petchey et al., 2008). We therefore believe that in many cases simpler models are likely to remain a valuable option for network inference, especially for bipartite networks such as those studied here.

Ultimately, the inference of interactions and the construction of potential food webs would extend the use and value of existing ecological census data. The inferred networks could then be used in ecosystem modeling or as null models to improve mechanistic understanding of species interactions (Bohan et al., 2021; Ma et al., 2019). The ecological census data we used here were two decades old and, so where suitable ecological census data exist, it could be possible to construct historical inferred networks from ecological census data, and for guilds where interaction data are difficult to obtain directly, such as many insectivorous predators. The inference of weighted interactions therefore greatly extends our potential to use ecological network approaches for times and places when the interactions were not directly sampled by reconstructing weighted ecological networks from the “ghosts of interactions past” (Bohan et al., 2017).

## CONFLICT OF INTEREST

The authors declare no conflicts of interest.

## AUTHOR CONTRIBUTIONS

**Michael J. O. Pocock:** Conceptualization (lead); Formal analysis (lead); Funding acquisition (supporting); Methodology (lead); Validation (equal); Writing‐original draft (lead); Writing‐review & editing (lead). **Reto Schmucki:** Resources (equal); Validation (equal); Writing‐review & editing (supporting). **David A. Bohan:** Funding acquisition (lead); Resources (equal); Validation (equal); Writing‐review & editing (supporting).

## Data Availability

Raw data on pitfall trap and seed rain data from the Farm Scale Evaluation of Genetically Modified Crops are available at: https://catalogue.ceh.ac.uk/documents/876358e4‐62f7‐4386‐99e1‐7d3eac223e03. Code to reproduce the analysis in this study and the trait data are available at http://doi.org/10.5281/zenodo.4252783.
